# Do urban open spaces provide refugia for frogs in urban environments?

**DOI:** 10.1371/journal.pone.0244932

**Published:** 2021-01-22

**Authors:** David Hutto, Kyle Barrett

**Affiliations:** Department of Forestry and Environmental Conservation, Clemson University, Clemson, South Carolina, United States of America; Universitat Autònoma de Barcelona, SPAIN

## Abstract

Urbanization is among the largest threats to wildlife populations through factors such as fragmentation, isolation, and habitat destruction. Urban open spaces, such as parks and golf courses, have the potential to provide wildlife with suitable habitat within an urbanized matrix. These refugia may be particularly important for amphibians, which represent one of the most endangered and least vagile vertebrate groups on earth. During the spring and summer of 2018, we conducted surveys to determine the presence of anurans at 51 wetland sites within the Piedmont ecoregion of South Carolina. Nearly one-third of these wetlands were located within urban open spaces, one-third in low development areas, and one-third in highly developed areas. Impervious surface and total road length surrounding the wetlands were measured at two scales, a core habitat scale (300 m) and average maximum migration scale (750 m), and we measured several within-wetland habitat variables. Urban Open Space wetlands had levels of surrounding impervious surface similar to High Urbanization wetlands at the larger scale and were intermediate between Low and High Urbanization wetlands at the smaller scale. The total length of road segments occurring within buffers (at both scales) surrounding our study wetlands was higher for Urban Open Space compared to Low and High Urbanization sites. Among the within-wetland variables measured, Low Urbanization sites had higher canopy cover and were more likely to have a terrestrial buffer zone relative to the other categories. Species richness decreased significantly as total road length increased among all wetlands. Wetland category was not a significant driver explaining species richness, but β-diversity was more variable among Urban Open Space wetlands than either Low or High Urbanization wetlands. Urban Open Space wetlands did not appear to increase suitability for anurans relative to High Urbanization wetlands. Urban Open Space wetlands had higher variability in species composition, which was perhaps attributable to the diversity among sites represented in the Urban Open Space category.

## Introduction

Undeveloped habitat is rapidly being replaced with urban infrastructure such as roads, buildings, houses, large paved areas, and other impervious surfaces [1, 2]. An increase in housing and building density has a negative effect on native species through habitat loss and reduction in habitat quality [2–6]. Decreases in native bird, arthropod, rodent, and amphibian species richness accompany urban density increases [7–11]. The long-term consequences of urbanization can be difficult to accurately predict, as negative responses of species to development may intensify over decades after initial development [3, 12].

As the size and density of urban development continues to increase, it is important to find areas that can serve as conservation refuges for species that might otherwise be negatively affected by urbanization. Urban open spaces (also referred to as green spaces) may offer areas of biodiversity conservation for native species within a matrix of otherwise unsuitable habitat [13, 14]. Urban open spaces are defined within this study as publicly accessible, managed outdoor spaces that are partly or completely covered by significant amounts of vegetation that exist primarily as semi-natural areas within an urban environment [15–17]. These areas may be public parks, community gardens, sports recreation zones (e.g. golf courses), or cemeteries. The habitat fragmentation, destruction, and isolation that occurs due to urbanization are all threats to biodiversity that urban open spaces can help mitigate [16]. Not only can urban open spaces help to preserve local biodiversity, their presence and the presence of the plant and animal species they can contain can also have positive psychological benefits to the people who utilize them [18, 19].

One group of animals that may particularly benefit from urban open spaces is amphibians. Their relatively small body sizes, low vagility, and small home ranges make them particularly vulnerable to localized habitat loss and urban impacts, and as such, ideal candidates for studies focusing on localized effects of urbanization. Amphibian species are at a higher risk of extinction than any other vertebrate class with nearly one-third (32%) of the world’s amphibians listed as threatened and 43% with declining populations as of 2004 [20–23]. These declines have been noted worldwide, with North America being no exception [24, 25]. While many studies have sought to evaluate amphibian responses to urbanization, few have evaluated how this group responds to small-scale habitat protection within a developed matrix [21, 26].

Urbanization and associated road densities can influence the movement of amphibians between suitable habitats and can increase the exposure of a wetland to pesticides, herbicides, and other chemical contaminants [4, 21, 27]. Additionally, urban development can increase wetland eutrophication through lawn fertilizer and chemical runoff and alter wetland hydrology [4, 27–29]. Urbanization also leads to an increase in the coverage of impervious surfaces across developed landscapes which, in turn, can contribute to habitat fragmentation by separating breeding wetlands from important upland habitats [29–32]. Not only do roads act as barriers between otherwise contiguous habitats, but traffic along these roads has a direct negative, and typically lethal, effect on anuran population [33, 34]. Frogs and toads may struggle to navigate even short distances (in relation to their overall dispersal abilities) in urbanized areas [32]. Factors such as vehicle collisions, exposure to runoff (salt, oil, etc.), noise, exhaust emissions, and vibrations can all affect anuran populations through either direct mortality or behavior interruptions [34, 35].

Despite the challenges that amphibians face in urban systems, urban open spaces can provide important habitat and habitat connectivity for anuran species, particularly those spaces containing wetlands or ponds [32, 36]. Not only can anurans benefit from these open spaces, but the open spaces can benefit from them. Tadpoles feed on algae and juvenile and adult anurans feed on insects which could reduce the need to utilize pesticides and herbicides, or to stock fish as an insect control method [28, 36].

While it is well-established that urban development can decrease the diversity and abundance of anurans, the efficacy of small-scale buffers around wetland habitats to bolster amphibian populations and diversity remains an open question [37]. Reviews of the literature indicate that wetland–breeding amphibians require anywhere from 300–750 m of upland habitat around breeding sites to carry out necessary life-history processes [31, 38–41]. Thus, we examined 51 wetlands along a rural-urban gradient in the Piedmont of South Carolina to assess anuran assemblages and compare wetlands and their surrounding upland habitat. Wetlands were assigned to one of three landscape categories: Low Urbanization, High Urbanization, and Urban Open Space. The first two categories were defined by the amount of development within a 750 m buffer. The third category was assigned to any wetland within an urban open space as defined above. Our objective was to evaluate anuran species richness and community composition as a function of within-wetland habitat features and the amount of urbanization within the surrounding landscape. We predicted that species richness would be highest at the “Low Urbanization” sites and that anuran assemblages at “Urban Open Space” sites would more closely resemble that of “Low Urbanization” sites given that urban open spaces may provide a buffer zone from urban influence.

## Methods and materials

### Ethics statement

The Institutional Animal Care and Use Committee (IACUC) within the Office of Research Compliance at Clemson University reviewed the study protocol and approved the research. Approval number: IACUC AUP2018-007.

Tadpoles were gently captured by long handled dip-net and immediately moved to a disinfected plastic bowl containing water from the same wetland. A small subset of tadpoles that were not able to be identified in the field were euthanized using MS-222 and taken to the lab for identification (1–2 tadpoles per unidentifiable species). We limited the number of animals to be euthanized to 50 per year. Adult and juvenile frogs/toads were gently captured by hand or long handled dip-net, identified to species, and immediately released.

### Study area and landscape characteristics

**Fig 1:**
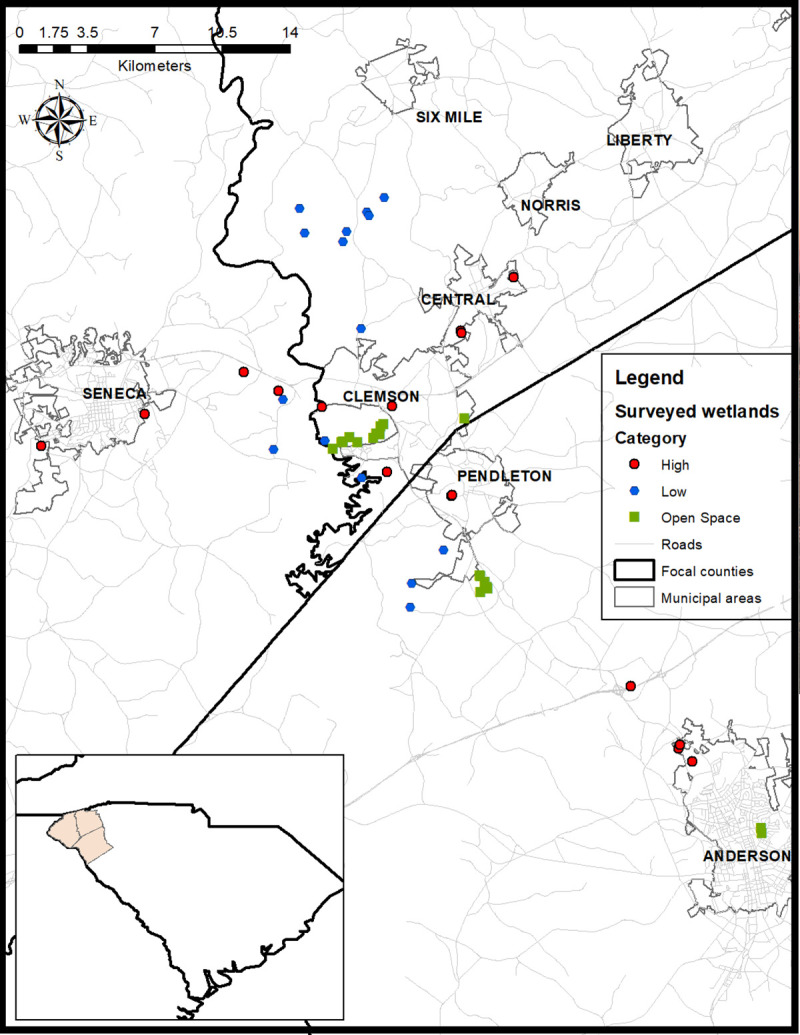
Map of the study area including sample sites. Red circles = High Urbanization; blue hexagons = Low Urbanization; green squares = Urban Open Space sites. The study area was within the South Carolina Piedmont Ecoregion and the focal counties are show on the inset. All layers used to build the image came from publicly available data. http://www.gis.sc.gov/data.html.

We used Google Earth to identify 73 wetland sites in Anderson, Oconee, and Pickens Counties in South Carolina for potential inclusion in our study. Throughout the late spring and summer of 2017, we assessed these sites for accessibility and differences in the amount of urbanized space surrounding each wetland and selected 51 finalist sites to include in our study (Fig 1). These wetlands are situated along a gradient ranging from rural to high urbanization (> 80% impervious surface surrounding the wetland). To address our study objectives, we placed each wetland site into one of three landscape categories: Low Urbanization, High Urbanization, or Urban Open Space, which included golf courses, parks, and gardens.

Wetland categorization was based on (1) whether the site was in an urban open space, and (2) if not, how much impervious surface surrounded the wetland within a 750-m buffer. Each wetland was delineated as a unique polygon in ArcMap (ESRI, Redlands, CA) using a satellite imagery base map combined with knowledge of the actual wetland boundaries determined by ground truthing. Two buffers were added around each wetland, one at 300 m based on a previous study that defined core habitat buffers around a wetland [38], and one at 750 m to encompass mean upland migration range as estimated by a review of anuran migration distances [41]. We labelled wetlands with impervious surface ≥ 30% within the 750-m buffer “High Urbanization”, whereas wetlands with < 30% impervious surface received the label “Low Urbanization.” We selected 30% impervious surface as a threshold because previous research has demonstrated amphibian declines within wetlands at or beyond this level of watershed development [37]. We used the 750-m buffer as our focal scale because it was inclusive of presumed maximum anuran movements around the wetland; within an anuran population, a buffer of 703 m was shown to include the 95% of localities for 262 individuals of 9 anuran species departing from breeding wetlands [41]. We gave the wetlands located in urban open spaces (as defined in the Introduction) the label “Urban Open Space.” Percentage of impervious surface surrounding wetlands in urban open spaces was not used to categorize these wetlands; nevertheless, the Urban Open Space category was primarily composed of sites with impervious surface coverage > 30% (only a single wetland was below this threshold with 25% impervious surface coverage). Urban Open Space wetlands encompassed several different management strategies. Most of the wetlands included in the Urban Open Space category were within golf courses (58%). These wetlands tended to be manicured ponds that were incorporated into the course as water hazards and received relatively constant management. The area around these ponds was mowed on a regular basis and the wetlands themselves were subjected to fertilizer and pesticide runoff. Among the golf course ponds, a few had natural edges along a portion of their banks. The remainder of the wetlands in the Urban Open Space category were within public parks and gardens (42%). These wetlands differed in the amount of management they received, both within and among parks. Some persisted in a nearly unmanaged state, whereas others had surrounding terrestrial vegetation routinely mowed. Our categorization scheme resulted in an allocation of 16 Low Urbanization, 16 High Urbanization, and 19 Urban Open Space wetlands.

We gathered developed land, impervious surface, and road map data layers at an image resolution of 30 m^2^ from the South Carolina Department of Transportation (SCDOT), and we calculated the amount of each within both buffer radii. The impervious surface layer included surfaces such as buildings, roads, and parking lots. Developed land and impervious surface were calculated as percent coverage within the buffer, while total road length was calculated as the sum of all road-length segments contained within a buffer. We examined the Pearson correlation coefficient for each pair of the above-mentioned landscape variables (percent developed land, percent impervious surface, and total length of road segments within a buffer). We removed percent developed land from all future analyses as it was highly correlated with impervious surface at both the 750 m and 300 m scale (0.92 and 0.85, respectively). We also removed impervious surface and road length at the 300-m scale from our analysis of species richness (see below) because the measures at this scale were highly correlated with the same measure at the larger (750 m) scale.

We also determined the distance from each of our study wetlands to the nearest body of water so we could model the effects of connectivity on the anuran assemblages therein. To do this, we obtained a layer of wetlands from the National Wetlands Inventory (NWI) that included all wetlands in South Carolina. We divided the polygons within the NWI shapefile into two separate categories within our analysis (riverine bodies and freshwater wetlands [freshwater emergent, forested/shrub, and ponds]) because we assumed these two wetland types may provide habitat to different assemblages of anurans. We calculated the straight-line distances from the edge of each of our study wetlands to the closest edge of each of the wetlands in these two categories. The distance between study sites was also measured to account for the possibility that study sites may be each other’s closest neighbor.

### Wetland site characteristics

At each site, we recorded habitat data (Table 1) during each daytime dip-net survey performed from March–July 2018 (additional information appears below under “Anuran Dip-net Surveys”). These data were collected to evaluate whether within-habitat features differed among the three wetland categories defined for this study. We measured wetland depth (m) at what we believed to be the deepest point in each of our study wetlands using a meter stick; wetlands deeper than the 1.2-m limit of the depth stick were given a depth of > 1.2 m. We obtained average organic layer depth by measuring the depth (cm) of the submerged organic layer at each dip-net stop and then averaging them together for each wetland. After leaf emergence (July), we measured canopy cover at each wetland by taking photos at each cardinal location using an iPhone 7 (Apple, Cupertino, CA) with a fisheye lens attachment (Amir, Shenzhen, Guangdong, China). For wetlands small enough (<0.01 ha) where multiple photos were not necessary to obtain canopy cover, we only took one photo at the center of each wetland. Conversely, at wetlands too large to obtain an accurate canopy reading with just four photos, we took a photo at every other dip–net site, which were selected at random to avoid biased sampling. Photos were taken from just above the water surface or the top of aquatic vegetation at each location. We then used the Gap Light Analyzer (Cary Institute of Ecosystem Studies, Millbrook, NY) to attain a percentage of canopy cover for each photo. We averaged the canopy cover across all photos for a wetland to produce its canopy cover estimate.

**Table 1:** Site and landscape characteristics collected for the 51 wetlands across three land use categories surveyed for anuran assemblage composition within the Piedmont of South Carolina.

	Method (unit)	High	Low	Urban Open Space
**Site characteristics**	** **	** **	** **	** **
Area	ArcMap delineation (ha)	0.28 (±0.53)	0.75 (±1.18)	0.48 (±0.96)
Wetland depth	Deepest point in wetland (m)	0.86 (±0.44)	1.03 (±0.31)	1.02 (±0.33)
Organic layer	Mean depth across all dip-net localities (cm)	4.2 (±2.7)	5.61 (±3.9)	5.15 (±3.7)
Canopy cover	Photo with fisheye lens (% cover)	24 (±21)	51 (±21)	26 (±25)
Aquatic vegetation	Visual estimate of emerged and submerged vegetation cover at dip-net localities (1 = 0–25%, 2 = 26–50%, 3 = 51–75%, 4 = 76–100%)	2.3 (±1.2)	2.2 (±1.1)	1.6 (±1.3)
Vegetation border	Assessed within 1-m of wetland boundary (present or absence)	75.0% with border	93.7% with border	53% with border
Fish	Visual assessment during dip-netting (presence/absence)	63% had fish	68.8% had fish	79% had fish
Hydroperiod	Visual assessment across surveys (permanent or temporary)	69% permanent	81% permanent	95% permanent
pH	Oaktron PCTSTestr	6.9 (±0.5)	6.8 (±0.4)	6.9 (±0.5)
Conductivity	Oaktron PCTSTestr (μS/m)	59.21 (±36.69)	55.78 (±37.30)	75.17 (±35.81)
**Landscape characteristics**	** **	** **	** **	** **
Distance to nearest river	Straight-line distance to feature calculated in ArcMap (m)	181.2 (±231.3)	108.4 (±166.1)	106.3 (±140.4)
Distance to nearest wetland	Straight-line distance to feature calculated in ArcMap (m)	141.3 (±198.0)	167.3 (±185.7)	84.0 (±89.5)

**Fig 2:**
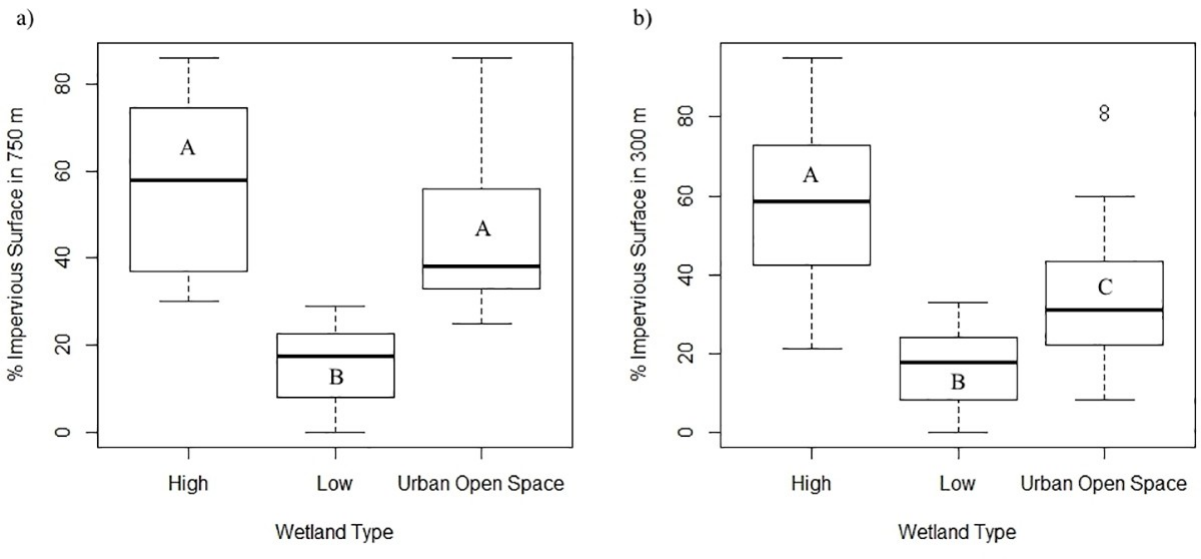
Box and whisker plots of percent of impervious surface within (a) a 750 m and (b) 300 m buffer of land surrounding wetlands in the South Carolina Piedmont ecoregion. Wetlands were divided into one of three categories (Low Urbanization, High Urbanization, and Urban Open Space) based first upon whether they occurred with an urban open space, and second, for those that were outside of such spaces, upon the percentage of impervious surface at the 750 m scale (*sensu* [41]). Dark bars represent median value, boxes represent the lower (25%) and upper (75%) quartiles, and whiskers represent the lowest and highest observed values up to 1.5 times the inter-quartile range. Values > 1.5 times the inter-quartile range are represented by open circles. Letters within boxes indicate pairwise significant differences determined through Tukey HSD test.

**Fig 3:**
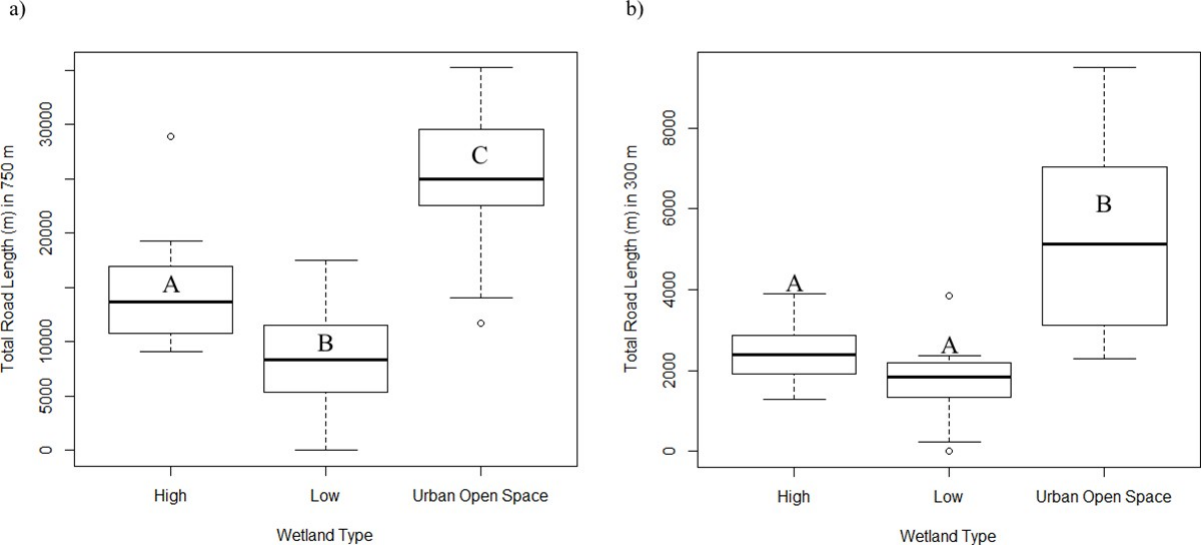
Box and whisker plots of total road length in meters within (a) a 750 m and (b) 300 m buffer of land surrounding wetlands in the South Carolina Piedmont ecoregion. Dark bars represent median value, boxes represent the lower (25%) and upper (75%) quartiles, and whiskers represent the lowest and highest observed values up to 1.5 times the inter-quartile range. Values > 1.5 times the inter-quartile range are represented by open circles. Letters within and outside boxes indicate pairwise significant differences determined through Tukey HSD test.

Significant figures reflect the resolution of measurement taken in the field or through GIS. For continuous variables, measures are presented as mean (±SD); binary variables are presented as percent of wetlands meeting a criterion. Impervious surface and road length are represented in Figs 2 and 3 respectively, so values for those variables are not presented here.

We surveyed vegetation cover within and around each wetland. We characterized aquatic vegetation cover by making visual estimates of the amount of emergent and submerged aquatic vegetation at each of our dip-net sampling points, and placing these estimates into categories (1 = 0–25%, 2 = 26–50%, 3 = 51–75%, 4 = 76–100%). A wetland was assigned the average value calculated across all sample locations. We noted the presence or absence of a border of herbaceous terrestrial vegetation within a 1-m buffer around wetlands. We assigned such a border as present if vegetation was at least 1 m in width and present around at least half of the wetland edge. Parris [39] and Puglis and Boone [36] suggest that terrestrial vegetation buffer zones around golf course wetlands may provide a more suitable habitat structure for anurans, providing shelter for metamorphs and adults as well as acting as sites for calling and amplexus. These buffer zones may also help mitigate the effects of applied chemicals [36].

Fish presence was assessed visually and via dip–net surveys. Wetlands where fish were not observed were assumed to not contain fish. We noted the presence or absence of water during all dip-net and call surveys to determine hydroperiod of each wetland during the sampling period (February-July). Wetlands that held water throughout the course of the study were given a value = 1 (permanent), and those that were dry at any point during the study were given a value = 0 (temporary). We measured pH, conductivity, and water temperature using an Oakton PCTSTestr^TM^ (Cole Parmer, Vernon Hills, IL) during each dip-net visit.

### Anuran dip-net surveys

Each wetland was surveyed three times from March–July, with the exception of five wetlands that only received two dip-net surveys due to weather and time constraints. Dip-net surveys lasted for a minimum of 20 minutes at each site [42]. Samples were taken across the entire wetland when possible, and were conducted at randomly chosen locations for lager wetlands. As there was a large variation in wetland size (size range = 0.002–8.71 ha), we increased maximum survey time by 10 minutes as wetlands doubled in size, up to a survey time limit of one hour. Therefore, small wetlands (size range = 0.002–0.12 ha), medium wetlands (size range = 0.13–0.24 ha), large wetlands (size range = 0.26–0.60 ha), and extra-large wetlands (size range = 0.64–8.71 ha) were surveyed for 20, 30, 40, and 60 minutes, respectively. As traversing many of those extra-large wetlands was not possible in a 60–minute time frame, we employed a sub-sampling technique. Specifically, we dip-netted within the wetland for 60 minutes, at 20 random points along the perimeter (selected within ArcGIS) for 3 minutes each. During this time we used a long handled, D-frame dip net to survey the shallow edge of wetlands and any vegetation present within these edges for larval anurans. Tadpoles were identified to species as closely as possible in the field and a small subset of those tadpoles that were unidentifiable were brought to the lab for further identification (IACUC AUP2018-007). We also recorded any visual encounters of adult/juvenile/and metamorphs, and egg masses (as they were identifiable) in order to assess species richness and diversity.

### Anuran call surveys

We conducted call surveys once per month from February–June for a total of five call surveys per wetland. These took place during the evenings beginning approximately 30 minutes after sundown and ending no later than 0100 the following morning. Surveys were conducted when temperatures were between 5.6°C and 30°C in order to maximize detection probability [43, 44]. In keeping with the protocol set forth by the North American Amphibian Monitoring Program (NAAMP), we conducted surveys when wind speed was less than or equal to a level 3 (8–12 mph) and did not conduct them during times of heavy rainfall as this could affect hearing ability. We spent five minutes actively listening at each site and recorded calls as an index of abundance as per NAAMP protocol (i.e. 1 = individuals can be counted; space between calls, 2 = calls of individuals can be distinguished but there is some overlap of calls, 3 = full chorus, calls are constant, continuous and overlapping). We used the Massachusetts noise index in order to account for ambient noise surrounding each wetland where (0 = no effect on sampling, 1 = slight effect on sampling, 2 = moderate effect on sampling, 3 = serious effect on sampling, 4 = profound effect on sampling) [43].

### Treefrog retreats

In order to account for adult treefrogs present at wetlands during dip-net surveys, we deployed white polyvinyl chloride (PVC) pipe retreats around wetlands. Each wetland received at least two retreats and we scaled up the number of retreats similar to the way we scaled up the time spent dip-net surveying at a wetland. Wetlands surveyed for 20 minutes received two, 30 minute wetlands received three, 40 minute wetlands received four, and the large wetlands actively surveyed for 60 minutes received five retreats for a total of 133 PVC retreats across all sites. Retreats were constructed similar to Boughton et al. [45]. All retreats measured 61-cm long and were 3.81-cm inside diameter. Each was fitted with a cap on the bottom to allow water to remain inside the retreat. A hole was drilled 25.4 cm from the bottom of the retreat to allow for excess water from rain events to drain out. We hung the retreats approximately 2 m above the ground in hardwood trees near the wetland edge using a small carabiner clip attached to a length of paracord that was tied around the trunk or limb of the tree. At wetlands where there were no suitable trees on which to hang retreats, they were secured to metal garden stakes at the ground level near the wetland edge. We deployed PVC retreats after the first round of dip-net surveys had been conducted (March) so retreats were only checked twice at each wetland during subsequent dip-net surveys. Each time retreats were checked, we identified any treefrog(s) within, cleaned out any debris, and replaced or added water as necessary.

### Data analysis

To evaluate the separation in landscape structure among the assigned land use categories, we used two separate one-way Analysis of Variance (ANOVA) tests followed by Tukey HSD pairwise comparison to assess differences in the amount of impervious surface and total road length surrounding wetlands by type (Low, High, and Urban Open Space) at both 750 m and 300 m. We conducted a Multivariate Analysis of Variance (MANOVA) followed by Tukey HSD pairwise comparison to evaluate differences in habitat variables and wetland category. Habitat variables included pH, conductivity, wetland depth, organic layer depth, canopy cover, area, and distances to the nearest river, and freshwater wetland (Table 1). We used a contingency table analysis followed by Pearson’s chi-squared test to account for differences in categorical environmental variables among wetland categories.

We also assessed whether development around our study wetlands had an influence on estimates of species richness at those sites. We estimated anuran species richness using the Chao index for incidence data on species detected during all survey types [46]. The Chao index estimates richness based on the number of rare species detected (i.e., species that were only detected once or twice during sampling). To test the hypothesis that anuran species richness is influenced by the amount of development, regardless of the assigned land use category, we performed linear regression on observed species richness against the percentage of impervious surface and total road length at the 750 m scale. We only examined one scale (750 m) because species richness was highly correlated between the two scales and the 750 m scale was our primary scale (see Methods, Study Area and Landscape Characteristics). We estimated β-diversity between wetlands using the Bray-Curtis distance metric on all anuran species detected. This metric ranges from 0 (identical species composition) to 1 (no shared species between sites). β-diversity estimates were subjected to an ANOVA followed by Tukey HSD pairwise comparison to assess differences among wetland categories.

We used Redundancy Analysis (RDA) to test for changes in community composition across wetland categories. RDA is a direct gradient analysis which summarizes linear relationships between components of response variables that are explained by a set of explanatory variables. Species data were summed across all dip–net visits and standardized on a scale of 0–10 with the maximum count of each species across all sites given a value of 10. Species with abundance estimates less than the maximum were assigned a standardized score (along the 0–10 scale) as a function of the highest abundance recorded for that species. For example, the site with the most individuals of Species A would be assigned a value of 10 for that species, and a site with half as many individuals of Species A would be assigned a 5. The RDA was constrained by wetland type to understand the percent of variation in species composition that could be explained by wetland category. We used Indicator Species Analysis on species detected during dip-net surveys to determine species associated with human land use as a function of wetland category. The indicator value index defines sets of species that distinguish predefined habitats [47]. Call survey data were not incorporated into either the RDA or the Indicator Species Analysis, since it was not possible to estimate abundance from the call surveys. All statistical analysis were performed in Program R Version 3.4.1 [48].

## Results

### Environmental and landscape variation among wetland categories

Landscape measures revealed the greatest distinction among wetland categories. There were significant differences among values of percent impervious surface surrounding wetlands within each category at both the 750 m (*F*_2,48_ = 27.67, *P*< 0.0008; Fig 2A) and 300 m scale (*F*_2,48_ = 19.08, *P* < 0.0001; Fig 2B). At the 750 m scale, percent impervious surface was significantly higher in High Urbanization sites relative to Low Urbanization wetlands (mean = 57.06 and 15.75, respectively; *P* < 0.00001) and in Urban Open Space wetlands relative to Low Urbanization sites (mean = 45.63 and 15.75, respectively; *P* < 0.00001). There was no significant difference in impervious surface between High Urbanization and Urban Open Space wetlands at the 750 m scale (Mean = 57.06 and 45.63, respectively; *P* = 0.11). At the 300 m core-habitat scale, the percentage of impervious surface was significantly higher at High Urbanization wetlands relative to both Urban Open Space (mean = 57.56 and 34.79, respectively; *P* = 0.002) and Low Urbanization wetlands (mean = 16.94; *P* < 0.001). Impervious surface was also significantly higher around Urban Open Space wetlands at the 300 m core-habitat scale relative to Low Urbanization wetlands (Mean = 34.79 and 16.94, respectively; *P* = 0.02).

Difference in road length surrounding all wetland types was also significantly different at both the 750 m scale (*F*_2,48_ = 40.88, *P* < 0.00001; Fig 3A) and the 300 m scale (*F*_2,48_ = 28.27, *P* < 0.00001; Fig 3B). At the 750 m scale, total road lengths were significantly higher at Urban Open Space sites relative to both High Urbanization sites (mean = 25,091 m/1.54 km^2^ and 14,460 m/1.54 km^2^, respectively; *P* < 0.001) and Low Urbanization sites (Mean = 25,091 m/1.54 km^2^ and 8,197 m/1.54 km^2^, respectively; *P* <0.001). Total road lengths at the 750 m scale were also significantly higher at High Urbanization wetlands than Low Urbanization sites (mean = 14,460 m/1.54 km^2^ and 8,197 m/1.54 km^2^, respectively; *P* = 0.008). Total road length at the 300 m core–habitat scale was higher around Urban Open Space wetlands than both High (Mean = 5,336 m/0.28 km^2^ and 2,429 m/0.28 km^2^, respectively; *P* < 0.001) and Low Urbanization wetlands (Mean = 1,665; *P* < 0.001). There was no significant difference in total road length between High and Low Urbanization wetlands at the 300 m scale (Mean = 2,429 m/0.28 km^2^ and 1,665 m/0.28 km^2^, respectively; *P* = 0.34).

Within-wetland measures of habitat were not statistically different among wetland categories (p > 0.05), except for canopy cover and terrestrial buffer immediately surrounding the wetland. Canopy cover was significantly different among the three wetland categories (*F*_2,48_ = 7.09, *P =* 0.002). Low Urbanization wetlands had the highest mean canopy cover (51.1%), which was significantly higher than High Urbanization wetlands and Urban Open Space wetlands (*F*_1,30_ = 12.97, *P* = 0.001 and *F*_1,33_ = 9.83, *P* = 0.003, respectively). Urban Open Space wetlands had a mean canopy cover of 26%, which was not significantly different from High Urbanization wetlands (24.3%; *F*_1,33_ = 0.04, *P* = 0.83). Contingency table analysis and Pearson’s chi-squared test revealed that the probability of a 1-m border of terrestrial vegetation around a wetland was not independent of wetland class. Specifically, Urban Open Space wetlands were less likely to have a terrestrial buffer zone (*x*^*2*^ = 7.44, *P* = 0.02). A 2x2 Chi-squared test on just the Low and High Urbanization wetland categories failed to reject the null hypothesis of independence between wetland category and the presence or absence of a terrestrial vegetation buffer zone (*x*^*2*^ = 0.94, *P* = 0.33).

### Anuran richness and diversity

During the 5–month sampling period at 51 wetland sites, we detected 12 anuran species. Of these 12 species, 3 are listed under the South Carolina Department of Natural Resources Wildlife Action Plan as species of priority [Pickerel Frogs (*Lithobates palustris*), Northern Cricket Frogs (*Acris crepitans*), and Upland Chorus Frogs (*Pseudacris feriarum*)] (South Carolina Department of Natural Resources, 2015). Green Frogs (*L*. *clamitans*) were the most common anuran, occurring at 84% of sites. An additional 5 species were also found at > 50% of sites [Fowler’s Toads (*Anaxyrus fowleri*) 57%, Gray Treefrogs (*Dryophytes versicolor*) 65%, American Bullfrogs (*L*. *catesbeianus*) 71%, Southern Leopard Frogs (*L*. *sphenocephalus*) 78%, and Spring Peepers (*P*. *crucifer*) 59%]. Three species were found to be present at over 50% of Urban Open Space wetlands: American Bullfrogs (63%), Southern Leopard Frogs (74%), and Green Frogs (79%; Table 2).

**Table 2:** Proportion of wetland categories occupied by anuran species during surveys (February–July 2018) in the South Carolina Piedmont ecoregion.

Taxon	Common Name	Wetland Type^a^		
		L	H	UOS
*Acris crepitans*	Northern Cricket Frog	0.69	0.44	0.37
*Anaxyrus americana*	American Toad	0.56	0.25	0.16
*Anaxyrus fowleri*	Fowler’s Toad	0.75	0.50	0.47
*Gastrophryne carolinensis*	Eastern Narrow-mouthed Toad	0.19	0.38	0.21
*Dryophytes cinerea*	Green Treefrog	0.50	0.56	0.32
*Dryophytes versicolor*	Gray Treefrog	0.81	0.81	0.37
*Lithobates catesbeianus*	American Bullfrog	0.75	0.75	0.63
*Lithobates clamitans*	Green Frog	0.88	0.88	0.79
*Lithobates palustris*	Pickerel Frog	0.19	0.06	0.00
*Lithobates sphenocephalus*	Southern Leopard Frog	0.81	0.81	0.74
*Pseudacris crucifer*	Spring Peeper	0.88	0.63	0.32
*Pseudacris feriarum*	Upland Chorus Frog	0.13	0.13	0.00

^a^L = Low Urbanization; H = High Urbanization; UOS = Urban Open Space

Wetlands were divided into one of three categories based on percentage of impervious surface at the 750 m scale (*sensu* [37] or their presence within an urban open space as defined by the study). Data presented here include detections from all survey types.

**Fig 4:**
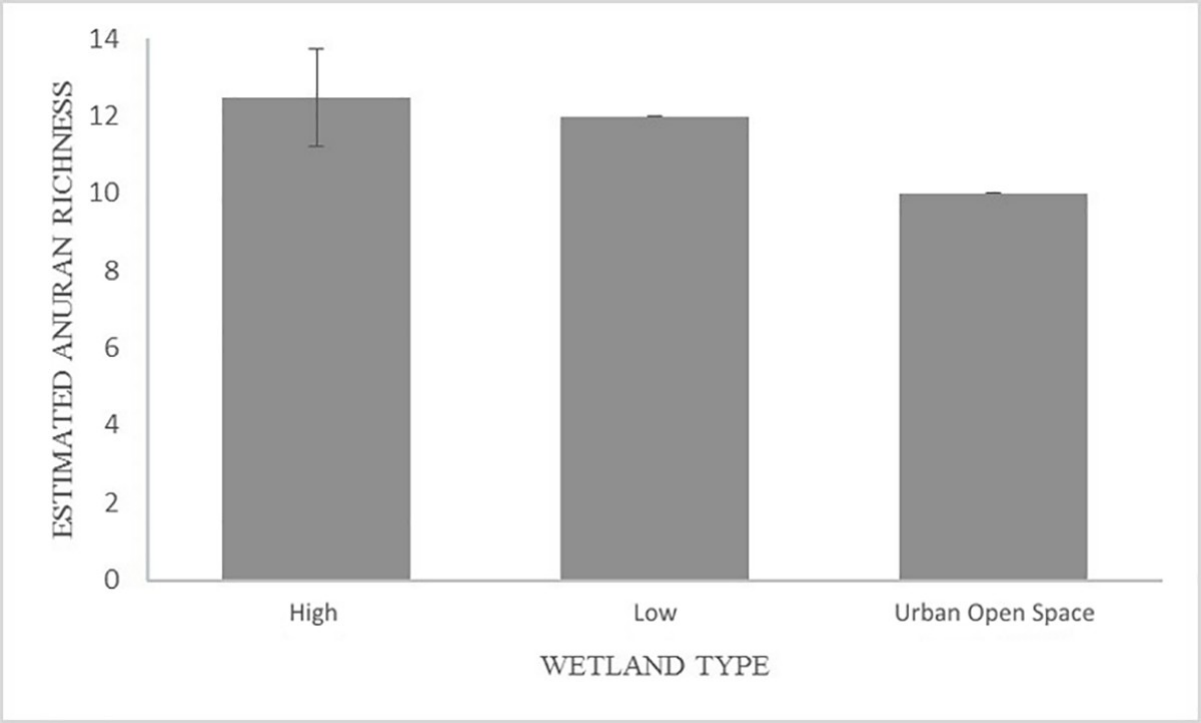
Chao estimated anuran species richness across three wetland types in the South Carolina Piedmont ecoregion.

**Fig 5:**
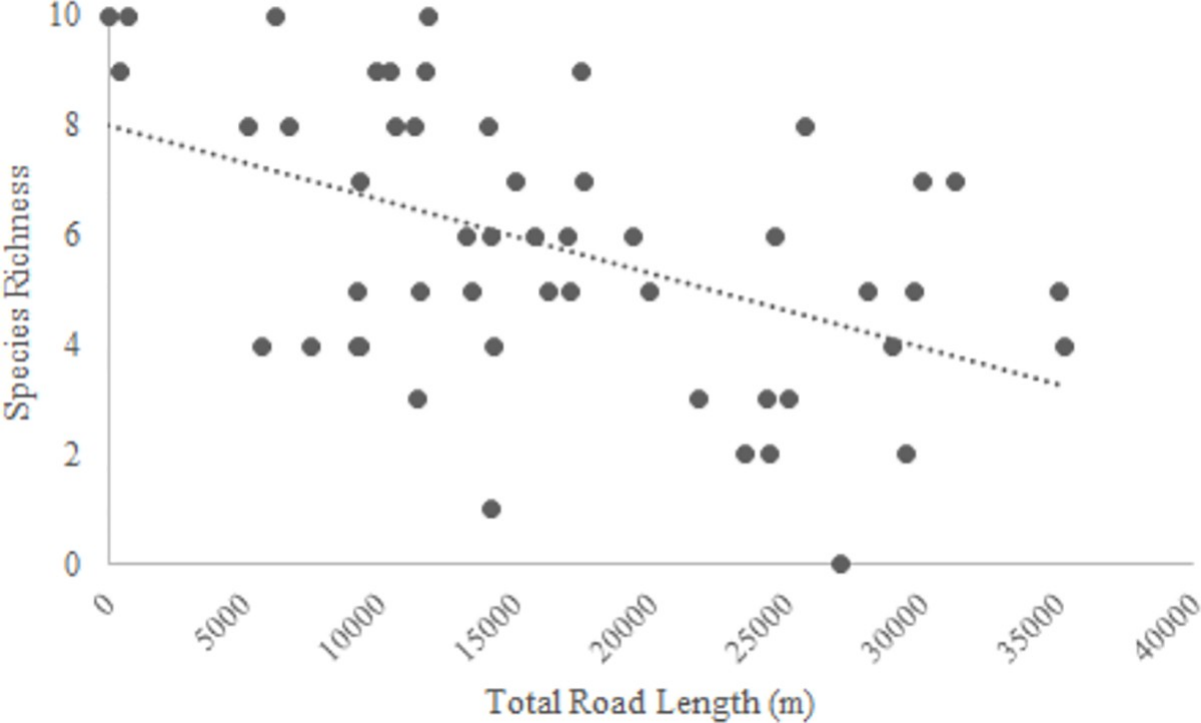
Observed anuran species richness declined significantly as total road length (m) increased in a 750 m buffer of land surrounding wetlands in the South Carolina Piedmont ecoregion.

**Fig 6:**
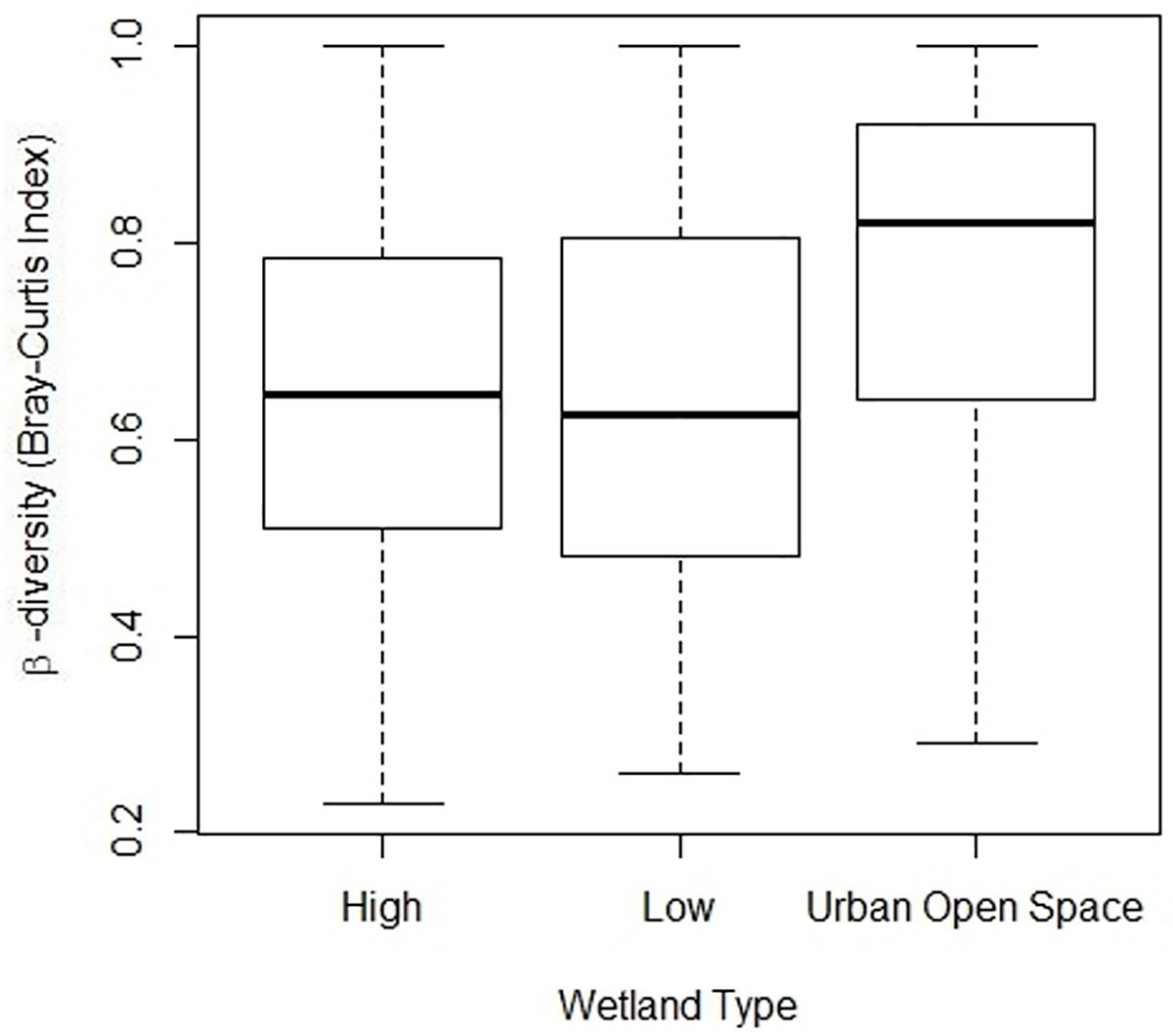
Box and whisker plot showing measurements of β-diversity (as measured by Bray-Curtis distance) among wetlands types in the South Carolina Piedmont ecoregion. Dark bars represent median value, boxes represent the lower (25%) and upper (75%) quartiles, and whiskers represent the lowest and highest estimate of β-diversity.

Observed species richness ranged from 0–10 species/site and was greatest in Low and High Urbanization sites (Fig 4); the Chao estimates of richness within categories varied little (< 1 species) from observed values. All 12 species were detected in Low Urbanization and High Urbanization wetlands, and 10 species were detected in Urban Open Space wetlands (Table 2). As the percentage of impervious surface within 750 m of the wetlands increased, species richness was unchanged (*β* = -0.27 ± 0.40, *P* = 0.50). As total road length within 750 m of a wetland increased, species richness decreased (*β* = -1.18 ± 0.40, *p* = 0.005; Fig 5). Analysis of β-diversity among wetlands within each class showed that Urban Open Space wetlands tend to be more variable from one another than either Low or High Urbanization wetlands (Fig 6). Indicator species analysis on species captured during dip net surveys revealed Spring Peepers (*P* < 0.001), American Toads (*P* = 0.002), and Northern Cricket Frogs (*P* = 0.01) were significantly associated with Low Urbanization wetlands. Indicator value is derived from two components that are each calculated between 0–1: specificity, which is highest when a species is only present at sites within a specific category and fidelity, which is highest when a species is present at all sites within a category. Fidelity values for Spring Peepers and Northern Cricket Frogs were relatively high (0.87 and 0.68, respectively) compared to that of American Toads (0.56). Specificity values were at least 0.60 for all three species but were particularly high for American Toads (0.79). No species were significant indicators for High Urbanization or Urban Open Space wetlands. The ordination of the entire species assemblage through RDA revealed that very little variation in structure (0.10) can be explained based on the three wetland land use categories.

## Discussion

Urban Open Space wetlands (wetlands within the urban matrix but immediately surrounded by greenspace) had lower species richness than wetlands in either High or Low Urbanization areas. While the lower amount of impervious surface surrounding an Urban Open Space wetland (as compared to a High Urbanization wetland) at the 300-m core habitat level is presumably beneficial to anurans, the presence of a higher total road length at this level may counteract such benefits by limiting dispersal and effectively favoring only a small subset of the available anuran community that is most tolerant of roads and fragmentation.

Development (measured by percent impervious surface coverage) surrounding Urban Open Space wetlands in our study area was statistically indistinguishable from High Urbanization wetlands at larger spatial scales; however, Urban Open Space wetlands were surrounded by levels of development intermediate between Low and High Urbanization sites at scales closer to the core habitat used by anurans (300 m). The total length of road segments surrounding Urban Open Space wetlands are higher at both the 300-m core habitat and average maximum migration scales than at either scale for Low and High Urbanization wetlands. This likely results from the networks of smaller roads that are abundant in areas supporting urban open spaces. Whereas the High Urbanization wetlands in our study may have more impervious surface surrounding them, less of that impervious surface was made up of roads relative to Urban Open Space wetlands. Urban open spaces are generally located in areas containing larger networks of smaller roads, and usually have grids of these small roads running through the open space themselves (walking and driving paths, cart paths, entrances and exits to the open space, etc.). While Urban Open Space wetlands may harbor less impervious surface in the surrounding upland habitat at the core level, this larger network of small roads can further fragment the landscape and act as a large barrier to dispersal of small-bodied animals [49, 50]. The negative relationship between road length and species richness further highlights the importance of roads in shaping anuran assemblages as opposed to an *a priori* site categorization.

High and Low Urbanization sites had similar total road lengths at the core habitat scale. On average most Low Urbanization wetlands were larger than High Urbanization wetlands and therefore contained a larger area within the 300 m buffer surrounding the wetland and, as such, had more land area within which roads could occur. Because buffers were measured from the edge of wetlands, not a centroid, larger wetlands at low urbanization sites simply had more surrounding area for roads to cross through, however road density would presumably remain lower than for High Urbanization wetlands and may be a helpful parameter to examine in future studies. The use of road length as the only metric of assessing road impacts may have limited our findings as all roads are assumed to be the same. Evaluating road densities, widths, total area, and traffic densities could help further explain the effects that networks of roads surrounding wetlands within and outside of urban open spaces have on anuran communities. Our impervious surface layer did, however, include roads in two dimensions (length and width) and ultimately may provide the most accurate view into the reality of road impacts.

Our Urban Open Space wetlands represented a diversity of open space types and management strategies, which may have contributed to some of the unexpected findings in our study. The majority of wetlands within golf courses received near constant management. These were mostly wetlands that contained less canopy cover than wetlands in parks and gardens and were manicured for ease of play along the course. Oftentimes the wetlands contained little aquatic or surrounding terrestrial vegetation and, as such, were unlikely to support diverse anuran assemblages. For example, we only observed an average of 4 anurans across the Urban Open Space wetlands within golf courses (n = 11), but a slightly higher average in Urban Open Space wetlands outside of golf courses (mean = 4.4). Given the small sample size, it is difficult to understand the low variation between these two wetland types, but our findings provide a good foundation for future study.

Wetlands within parks were managed on a continuum from heavily managed zones with little to no aquatic vegetation and virtually no surrounding terrestrial vegetation to sites with no management intervention within the wetland buffer. A difference in management strategies may also help explain the greater species richness observed at High Urbanization sites as compared to Urban Open Space sites. Overall, wetlands within the High Urbanization category tended to be relatively natural wetlands or storm water drainages that received very little management. A few sites in this category received regular management to minimize surrounding terrestrial vegetation, but many were left unmanaged. This lack of management may lead to a more suitable habitat for anurans within urban matrices, potentially driving a higher species richness than some more heavily managed wetlands. While the influence of a border of herbaceous vegetation around the wetland was not an explicit a priori hypothesis, our data do suggest the presence of such vegetation is associated with sites where more species were detected. Sites with and without a vegetated border had a mean species richness of 6.3 (SE = 0.4) and 4.5 (SE = 0.6), respectively. Based on this data, the planting and/or maintaining of a vegetated border around wetlands may aid in bolstering a sites ability to support a diverse anuran population. The ability of wetlands in highly urbanized areas to support relatively high anuran species richness should not be overlooked as many such wetlands may be important for anuran conservation [51].

While we did not explicitly test the relative influence of within-wetland vs. landscape-scale variables on anuran communities, both factors varied among our three habitat types and may have contributed to the resulting assemblage composition. Fragmentation of upland habitats by urban development is known to negatively influence some anuran species [4, 52]. Nevertheless, it is reasonable to assume that not all forms of impervious surface are equivalent in terms of the effects on anuran population response. Large retail or industrial buildings that are accompanied by expansive parking lots presumably have stronger negative effects on population growth relative to low-density residential development. Our analysis did not tease out these differences, and more work remains to be done on this front. Lower levels of amphibian richness have been described in other settings with fragmented landscapes; these findings elsewhere may have relevance and applicability to the low levels of anuran species richness found in conjunction with road length in this study [11, 23, 28]. Those findings, along with ours, underscore the likelihood that amphibian assemblage trends are probably better understood in a gradient context rather than categorically.

Generally, more species were found in the Low or High Urbanization wetlands. These results may be due, in part, to the inclusion of species-deficient golf-course wetlands in the Urban Open Space category. Measures of β-diversity among wetland types also showed that wetlands located in urban open spaces tend to be more variable in species composition from one another than those in Low or High Urbanization areas. The variability within Urban Open Space wetlands may result from variation in habitat features at these sites (such as the presence or absence of a vegetated buffer zone). Reviews of the literature show that the isolation of urban open space from other natural areas can lead to a shift in species assemblage as generalist species (urban adapters) continue to colonize whereas sensitive and specialist species disappear [14]. Studies have also shown that an increase in the amount of human recreation in an area can have negative effects, directly and indirectly on reptile and amphibian species [53, 54]. Nevertheless, research has shown that in the presence of key habitat features, for example, a 1-meter vegetated border as assessed in this study, certain amphibians will persist even in urban environments [4, 32, 55, 56].

Body size and dispersal ability may also drive the species of anurans present at some Urban Open Space sites. Those species that inhabit higher proportions of Urban Open Space wetlands tend to have larger overall body sizes and they exhibit post-metamorphic life stages that are primarily associated with aquatic habitats such as Green Frogs, American Bullfrogs, and Southern Leopard Frogs, though this is not always the rule [4, 57]. Though some of these larger-bodied species may be particularly vagile among anurans, alterations to wetlands and surrounding habitat (such as a reduction in ground vegetation) as well as human disturbances may limit their movement and dispersal ability [58]. The three ranids also tend to hibernate under water or enter into dormancy near water during cold months, allowing them to persist within or closely nearby a wetland throughout their life [59, 60]. The extensive use of upland habitats by species such as Gray Treefrogs, Spring Peepers, and American Toads may be hindered by the higher presence of roads surrounding their breeding wetlands and may eventually drive them out of a wetland altogether [4, 39]. The reduction of suitable upland habitats surrounding wetlands may also negatively affect these species [4]. A reduction in successful migration rates of juveniles and adults between ponds or to upland habitats can, over time, lead to a decline in the numbers of anuran species present within these environments.

## Conclusion

We did not detect significant differences in community structure among wetland types (Low Urbanization, High Urbanization, and Urban Open Space), though we did see lower species richness within Urban Open Space wetlands and with increasing levels of total road length surrounding wetlands. The negative relationship between species richness and road length (Fig 4) reflects the findings of others. Such declines likely are a result of landscape alterations, decreased wetland/upland connectivity, and decreased wetland availability [4, 11, 39, 61]. The percentage of impervious surface surrounding the wetlands within the study provided the basis for their assignments into either the Low or High Urbanization categories; the assigning of wetlands into the Urban Open Space category was subject to their inclusion within a predefined urban open space. The fact that we found a negative relationship between species richness with increasing road length, but no discernable trend among wetland categories, may indicate that responses to development are continuous rather than threshold-like. Further, our post hoc assessment of sites with and without a terrestrial buffer suggests the presence of vegetation around the wetland may facilitate the presence of more species regardless of the surrounding land use.

If anuran diversity is to be maximized and maintained, better understanding of key factors that drive anuran populations within urban environments is imperative. Management strategies may consider prioritizing wetlands that are already situated in areas with few roads in the surrounding landscape. Minimizing the density of roads surrounding urban open spaces may serve to benefit anurans as they move within and between open spaces and the surrounding landscape. Installing underpasses at strategic road locations may also aid in improving connectivity in Urban Open Spaces and increasing successful anuran dispersal and migration. Additionally, understanding how the utilization of differing management strategies within urban open spaces affects anurans may further enhance the conservation potential for these spaces. Higher observed species richness within High Urbanization wetlands may be attributed to the lack of management observed at the majority of these sites throughout the course of the study compared to Urban Open Space sites, however further empirical study is required to test this hypothesis. Regardless of the underlying mechanisms, we documented that developed areas can support an anuran assemblage that is similar to less developed regions. This finding demonstrates the potential conservation value of all wetlands; however, many questions regarding long-term population status of the species within these urban assemblages remain to be addressed.

## Supporting information

S1 Dataset Sites x species data. (CSV)

S2 Dataset Sites x environmental data. (CSV)

S3 Dataset Sites x species occurrence data. (CSV)
